# Association between educational attainment and risky sexual behaviour among Ghanaian female youth

**DOI:** 10.4314/ahs.v23i1.32

**Published:** 2023-03

**Authors:** Frederica Jackson, Zelalem T Haile

**Affiliations:** 1 Department of Social and Public Health, College of Health Sciences and Professions, Ohio University, Grover Center W347, Athens, OH 45701, USA; 2 Department of Social Medicine, Ohio University Heritage College of Osteopathic Medicine, 6775 Bobcat Way, Room 450, Dublin, OH 43016, USA

**Keywords:** Ghanaian youth, risky sexual behavior, educational attainment

## Abstract

**Background:**

Ghanaian female youth aged 15-24 years are especially vulnerable to sexually transmitted infections (STIs) compared to their male counterparts.

**Objectives:**

This study examined the association between educational attainment and risky sexual behavior among Ghanaian female youth.

**Method:**

A cross-sectional study was conducted using a nationally representative sample of youth from the 2014 Ghana Demographic and Health Survey (n=1702). The outcome of interest was risky sexual behavior, defined as having last intercourse with a non-marital partner without condoms. Descriptive and inferential statistical tests were utilized.

**Results:**

Overall, the prevalence of risky sexual behavior was 66.9%. In the bivariate analysis, risky sexual behavior differed by level of education. Compared to women with a college-level education, a significantly higher proportion of women with secondary education reported risky sexual behavior (72.9%), followed by those with primary education (65.7%) (p<0.001). In the multivariable-adjusted model, there was a significant interaction between education and household wealth index (P =0.030) and between education and the type of place of residence (P =0.045) on risky sexual behavior.

**Conclusion:**

Culturally appropriate and targeted interventions are warranted to increase condom use among these subgroups and curb the rising rates of STIs among Ghanaian female youth.

## Introduction

Sexually transmitted infections (STIs) have been a public health issue for decades. With the emergence of HIV and AIDS in the early 1980s, STIs have claimed millions of lives. The World Health Organization (WHO) reports that an average of 1 million STIs is acquired daily worldwide^1^. Again, about 376 million new infections occur annually, with 1 of 4 most common STIs: chlamydia, gonorrhea, syphilis, and trichomoniasis^1^. Other forms of STIs include HIV, hepatitis B, herpes simplex virus (HSV or herpes) and human papillomavirus (HPV), the predominant mode of transmission is through sexual contact (vagina, anal, oral sex) and blood or blood products^1^. In the last decade, AIDS deaths have declined by 33%^2^,^3^; however, HIV incidence and prevalence continue to rise steadily^4^. Despite ongoing prevention efforts, STI infections continue to be a significant health problem, especially in sub-Saharan Africa. Young people, particularly females aged 15-24 years, are reported to be most at risk; existing research suggests that high levels of risky sexual behavior are the major contributor to high STI infections in the region.^5^–^7^. Risky sexual behavior has been defined as having sexual intercourse with at least one non-spousal partner without using a barrier method^8^.

Ghana is no exception to the alarming rates of STI infection brought on by risky sexual behaviours^9^,^10^ and existing research attempts to identify its driving factors. For instance, Ohene & Akoto (2008) report barriers in accessing condoms as a reason for failure to use condoms in the last sexual encounter^11^. Additionally, other researchers have indicated that youth reported lower susceptibility to infection and self-efficacy to use condoms as prime reasons for engaging in risky sexual behavior^12^.

To our knowledge, few studies have tried to examine if there is an association between educational attainment and risky sexual behavior among Ghanaian youth. One cross-sectional study that examines reproductive health risk and protective factors among unmarried youth in Ghana evaluated the influence of school attendance on the initiation of sex and the number of sexual partners^13^ but failed to report differences by educational levels. The current study explores the association between educational attainment and risky sexual behavior among Ghanaian female youth aged 15-24 years. Specifically, the study compares reported risky sexual behaviours between youth who have less than secondary education and youth who have received college-level education. We hypothesize that a higher level of education is associated with reduced engagement in risky sexual behaviours. We further tested whether this association differs by other characteristics, including household wealth index and type of place of residence.

## Ethical considerations

The 2014 Ghana Demographic and Health (GDHS) Survey received government permission, used informed consent, and assured participants of confidentiality. The current study was considered exempt from a full review by the Institutional Review Board of the authors institution because it was based on an anonymous public use of a secondary dataset with unidentifiable information on the survey participants.

## Methods

### Data

The current study utilizes data from the 2014 Ghana Demographic and Health Survey, a nationally representative cross-sectional survey that provides up-to-date estimates and a snapshot view of the current state of health of the Ghanaian population 14. The GDHS is nationally and privately funded and includes a total of 3 questionnaires: household questionnaire, man's questionnaire and woman's questionnaire^15^,^16^.

### Sample

Using a stratified two-stage cluster sampling technique, 12,831 households were selected and 9,396 women aged 15-49 years were interviewed. The current study was restricted to women aged 15-24 years (n=3327). We excluded women who had never had sex (n=1211) and women with missing data (n=414) on risky sexual behavior and other covariates included in the multivariable model. The final sample consisted of 1702 participants^16^.

### Measurement

#### Dependent Variable: Risky Sexual Behavior

Risky sexual behavior for this study was defined as having sex with one or more non-spousal partners without condoms. This was assessed by combining responses to the following survey questions: i) *When was the last time you had sexual intercourse?* Response options included number of days, weeks, months and years ii) *“What was your relationship to this person with whom you had sexual intercourse?”* Response options included: husband, live-in partner, boyfriend not living with the respondent, casual acquaintance, client/prostitute, other iii) *“Was a condom used every time you had sexual intercourse with this person?”* In the current study, risky sexual behavior (RSB) was coded as “Yes” if a woman reported having sex with at least one non-spousal partner/s without condoms and “No” otherwise.

#### Independent Variable: Educational Attainment

Educational attainment was assessed by asking participants, *“Have you ever attended school?”* If the response was affirmative, subsequent questions included *“What is the highest level of school you attended?”* Response options included: Primary, middle, Junior Secondary School/Junior High School, Secondary, Senior Secondary School/Senior High School, or higher. Subsequent outcomes of interest included *“What is the highest grade you completed at that level?” “Are you currently attending school at any level?”* In the current study, educational attainment was categorized into 4 sub-groups: No education, primary education, secondary, and higher.

#### Covariates

Based on prior studies^8^,^10^,^11^, potential confounders examined in the current study include age, marital status, household wealth index, type of residence, total lifetime number of sex partners, religion, sex of household head, media exposure, history of sexually transmitted infections, and age at onset of sexual activity.

### Statistical Analysis

Frequencies and proportions were used to describe the characteristics of the study sample. The Rao-Scott Chi-square test was used to examine differences in the proportions of risky sexual behavior by educational attainment and each covariate examined in the current study. Multivariable-adjusted logistic regression analysis was carried out to examine the independent association of educational attainment and risky sexual behavior. Both unadjusted and multivariable-adjusted odds ratios (OR) and corresponding 95% confidence intervals (CI) were reported. All variables were retained in the multivariable model regardless of statistical significance in bivariate analysis. Multicollinearity was checked using the variance inflation factor (VIF). Using a conservative threshold VIF value of 4, no collinearity was detected. Pair-wise interaction terms between educational attainment and each covariate were included in the multivariable model to assess subgroup differences in the association between educational attainment and risky sexual behavior. P-value <0.05 was considered statistically significant. Survey design elements were applied in all analyses to account for complex survey design. All analyses were performed using SAS® 9.4.

## Results

Table 1 presents the characteristics of the study sample. Of the 1702 sexually active women, 66.9% reported engaging in risky sexual behavior, 3.9% had greater than high school education, 58.1% were never married, 81.1% were Christians, 52% lived in rural areas and 64.5% reported having their first sexual encounter before the age of 18. Additional characteristics of the study sample are shown in table 1.

**Table 1:** Descriptive statistics of the study sample (N=1702)

	Overall n (wt. %)
**Age**	
15–17	552 (31.1)
18–24	1150 (69.9)
**Marital status**	
Single/ never married	949 (58.1)
Married/Living together	699 (38.1)
Div/sep/widowed	54 (3.8)
**Educational Attainment**	
None	218(10.8)
Primary	338 (18.2)
Secondary	1088 (67.1)
Higher	58 (3.9)
**Household wealth index**	
Poor	785 (37.0)
Middle	414 (25.0)
Rich	503 (38.0)
**Religion**	
Christian	1319 (81.1)
Muslim	312 (14.6)
Other	71 (4.3)
**Head of household**	
Male	1054 (58.2)
Female	648 (41.8)
**Residence**	
Urban	755 (48.0)
Rural	947 (52.0)
**Media exposure**	
No	133 (6.5)
Yes	1569 (93.5)
**Number of lifetime sex partners**	
One	817 (44.0)
More than one	885 (56.0)
**Age at first sex**	
Before 18	1113 (64.5)
18 or older	589 (35.5)
**Previous STI infection**	
No	1563 (91.3)
Yes	139 (8.7)
**Risky sexual behavior**	
No	680 (33.1)
Yes	1022 (66.9)

Abbreviations: Wt.%: Weighted percent

Table 2 displays the results of the bivariate analysis. A greater proportion of participants with secondary education reported engaging in risky sexual behavior (72.9%), followed by those with primary education (65.7%), college (54.0%) and no education (36.5%), respectively (p<0.001). Other variables that were significantly associated with risky sexual behavior include age, marital status, household wealth, religion, head of household, and the number of lifetime sex partners. Women who reported engaging in risky sexual behavior were more likely to be 20-24 years old, not currently married/living together, Christian, reside in middle household wealth index, reside in female-headed households and had more than one sexual partner in their lifetime.

**Table 2:** Characteristics of the study sample by Risky Sexual Behavior (N=1702)

	Risky Sexual Behavior				
				
	No n (Wt. %)	Yes n (Wt.%)	p	Unadjusted OR (95% CI)	p
**Age**			<0.001		
15–19	167 (23.6)	385 (76.4)		Reference	
20–24	513 (37.4)	637(62.6)		0.52 (0.39, 0.68)	<0.001
**Marital status**			<0.001		
Single/ never married	230 (19.9)	719 (80.1)		Reference	
Married/Living together	439 (54.6)	260 (45.4)		0.21 (0.15, 0.28)	<0.001
Div/sep/widowed	11 (19.0)	43 (81.0)		1.05 (0.44, 2.56)	0.898
**Educational attainment**			<0.001		
None	146 (63.5)	72 (36.5)		0.49 (0.22, 1.10)	0.083
Primary	135 (34.3)	203 (65.7)		1.63 (0.79, 3.36)	0.182
Secondary	371 (27.1)	717(72.9)		2.29 (1.15, 4.54)	0.018
Higher	28 (46)	30 (54)		Reference	
**Household wealth index**			0.002		
Poor	354 (39.1)	431 (60.9)		Reference	
Middle	132 (25.7)	282 (74.3)		1.86 (1.32, 2.62)	0.004
Rich	194 (32.1)	309 (67.9)		1.36 (0.98, 1.88)	0.064
**Religion**			<0.001		
Christian	474 (29.0)	845 (71.0)		Reference	
Islam	181 (55.5)	131 (44.5)		0.33 (0.23, 0.47)	<0.001
Other	25 (35.0)	46 (65.0)		0.76 (0.34, 1.70)	0.500
**Head of household**			<0.001		
Male	489 (40.0)	565 (60.2)		Reference	
Female	191 (23.9)	457 (76.1)		2.11 (1.61, 2.75)	<0.001
**Residence**			0.181		
Urban	286 (30.9)	469 (69.1)		1.22 (0.91, 1.62)	0.182
Rural	394 (35.2)	553 (64.8)		Reference	
**Media exposure**			0.599		
No	54 (35.6)	79 (64.4)		Reference	
Yes	626 (32.9)	943 (67.1)		1.13 (0.72, 1.78)	0.601
**No. of lifetime sex partners**			0.031		
One	360 (36.6)	457 (63.4)		Reference	
More than one	320 (30.4)	565 (69.6)		1.32 (1.02, 1.71)	0.035
Age at first sex			0.523		
Before 18	249 (34.3)	340 (65.7)		1.09 (0.84, 1.42)	0.524
18 or older	431 (32.4)	682 (67.6)		Reference	
Previous STI infection			0.113		
No	637 (33.8)	926 (66.2)		Reference	
Yes	43 (25.9)	96 (74.1)		1.46 (0.91, 2.35)	0.119

Abbreviations: Wt.%: Weighted percent.

P- values are derived from Rao-Scott Chi-square test

In the multivariable model, we found a significant interaction between education and household wealth index (P for interaction =0.030) and between education and the type of place of residence (P for interaction =0.045). Table 3 and Table 4 present the association between educational attainment and risky sexual behavior stratified by household wealth index and type of residence, respectively. Among youth who reside in rich households, compared to women with a college education, the odds of risky sexual behavior were higher in women with secondary education (OR, 3.11; 95% CI 1.35 – 7.17; P =0.008) (Table 3).

**Table 3:** Association between educational attainment and risky sexual behavior stratified by household wealth index (N=1702)

	Poor (n=785)		Middle (n=414)		Rich (n=503)	
			
	OR (95% CI)	P	OR (95% CI)	p	OR (95% CI)	p
**Educational attainment**						
None	0.42 (0.05, 3.45)	0.421	3.2 (0.43, 24.25)	0.257	0.83 (0.12, 6.03)	0.856
Primary	0.95 (0.12, 7.49)	0.962	4.24 (0.54, 33.07)	0.167	1.98 (0.73, 5.39)	0.178
Secondary	0.87 (0.11, 6.72)	0.895	2.76 (0.4, 19.0)	0.301	3.11 (1.35, 7.17)	0.008
Higher	Reference		Reference		Reference	

Models adjusted for include age, marital status, head of household, religion, place of residence, age at first sex, media exposure, previous STI infection and number of lifetime sex partners.

**Table 4:** Association between educational attainment and risky sexual behavior stratified by place of residence (N=1702)

	Urban (n=755)		Rural (n=947)	
		
	OR (95% CI)	P	OR (95% CI)	P
**Educational attainment**				
None	1.85 (0.57, 6.01)	0.307	1.43 (0.37, 5.57)	0.605
Primary	3.94 (1.46, 10.61)	0.007	2.89 (0.86, 9.77)	0.087
Secondary	3.25 (1.40, 7.53)	0.006	3.26 (0.98, 10.91)	0.055
Higher	Reference		Reference	

Models adjusted for include age, marital status, head of household, religion, wealth index, age at first sex, media exposure, previous STI infection and number of lifetime sex partners.

Among urban youth, compared to women with a college education, the odds of risky sexual behavior were higher among women with primary education (OR, 3.94; 95% CI 1.46 – 10.61; P=0.007) (Table 4) and secondary education (OR, 3.25; 95% CI 1.40- 7.53; P=0.006) (Table 4).

## Discussion

In a large, nationally representative sample of Ghanaian female youth, household wealth index and type of place of residence moderated the association between educational attainment and risky sexual behavior. The odds of risky sexual behavior were higher among participants with primary and secondary level education compared to those with college-level education. The observed association was independent of potential confounders. However, the association was present only in subgroups of women residing in urban and rich households.

The present findings are supported by recent studies that found a relationship between higher levels of educational attainment and lower sexual risk-taking behaviours among the youth. Particularly, documented research has found higher forms of educational attainment as a protective factor for risky sexual behavior among sexually active females 10, 11, 17. Zuilkowski & Jukes, in a systematic review of the literature, found that higher levels of education were significantly associated with higher rates of condom use among sexually active female youth in 23 out of 44 studies reviewed 17.

In the current study, we observed that women from high-income households were more likely to engage in risky sexual behavior. Similar associations were found in a study conducted by Odimegwu et al. 18 using DHS data from other African countries, which revealed that women from richer households were more likely to engage in high sexual risk-taking behavior 18. The finding adds to the existing evidence that suggests that risky sexual behavior is not solely a consequence of poverty but rather a myriad of other intersecting factors that go beyond economic standing. Some plausible mechanisms that may explain the observed association include the fact that women from richer households may tend to travel more frequently and, as such, may not always readily have condoms available to them 18. Researchers have also found that youth from well-to-do households have more disposable income and thus are more likely to misuse drugs leading to high forms of sexual risk-taking behaviours^19^,^20^. Further, these youth are more likely to have access to erotic material through social media which has been linked to the prevalence of a hook-up culture in this population 21, 22. For instance, Carmack & Rodriguez (2020) found that higher Facebook usage among female study participants (17- 23 years) was associated with having concurrent sexual partners and the pursuit of hookups^21^. Moreover, existing research indicates that female youth who frequently use social media are relatively more likely to share sexually explicit texts, images and videos with partners and/or strangers; such acts may lead to sexual risk-taking among female youth 23, 24.

While some previous studies have found significantly higher rates of risky sexual behavior among rural youth^25^ the current study found high levels of risky sexual behavior among urban women. Previous research shed light on patterns of risk-taking behavior by place of residence and confirms that urban women, compared with rural women, had relatively higher odds of engaging in risky sexual behavior 18, 26. Idowu and colleagues provide further supporting evidence through their study on secondary school youth residing in urban areas in Nigeria 26. The authors report significantly higher rates of unsafe sexual practices among secondary school youth and hypothesize that having sex among this population is often casual and unplanned 26.

In summary, our study shows a significant association between educational attainment and risky sexual behavior. This association was moderated by the household wealth index and the type of place of residence. Among women residing in rich households as well as among rural youth, compared to women who had college-level education, lower levels of educational attainment increased the likelihood of risky sexual behavior. This study sheds light on the importance of higher education in curbing the rising rates of STI and HIV infections in the country. Furthermore, findings reveal existing gaps in the marketing strategy and promotion of condom use targeted towards women of lower educational attainment.

The current study has some strengths. The sample was composed of large nationally representative participants collected using rigorous methods. The availability of several variables for multivariable adjustment is an additional strength of the study. The study, however, is not without its weaknesses. Our analysis was limited to variables available in the 2014 GDHS. Important variables such as participant perceptions and condom negotiation and barriers in accessing condoms, including availability, societal stigma and distance to the nearest pharmacy, were not captured in the 2014 GDHS. Additionally, because DHS data is self-reported, it is susceptible to information bias. Moreover, the questions based on condom use during the

## Conclusion

Our results showed a positive association between lower educational attainment and risky sexual behavior in a large, nationally representative, sample of Ghanaian female youth. The unique nature of our study is the report that the association between educational attainment and risky sexual behavior was present only in urban and rich household residents. Risky sexual behavior is preventable. Findings from the study are essential in developing targeted interventions to increase safe sex practices among urban women with primary and secondary level education and for Ghanaian female youth from wealthier households. Future strategies should employ a multi-dimensional perspective that examines the social, physical, cultural, community and interpersonal relationships that may be moderating the association between risky sexual behavior and educational attainment among these subgroups. The findings from this study can be used as a framework for a theory-based investigation in the future.
